# Role of NLRP3 inflammasome and oxidative stress in hepatic insulin resistance and the ameliorative effect of phytochemical intervention

**DOI:** 10.3389/fphar.2023.1188829

**Published:** 2023-06-30

**Authors:** Isabela Jesus de Deus, Ana Flávia Martins-Silva, Miliane Martins de Andrade Fagundes, Sílvia Paula-Gomes, Fernanda Guimarães Drummond e Silva, Larissa Leandro da Cruz, Aline Rezende Ribeiro de Abreu, Karina Barbosa de Queiroz

**Affiliations:** ^1^ Laboratório de Nutrição Experimental, Programa de Pós-Graduação em Saúde e Nutrição, Escola de Nutrição, Universidade Federal de Ouro Preto, Ouro Preto, Brazil; ^2^ Departamento de Alimentos, Programa de Pós-Graduação em Saúde e Nutrição, Escola de Nutrição, Universidade Federal de Ouro Preto, Ouro Preto, Brazil; ^3^ Laboratório de Bioquímica e Biologia Molecular, Programa de Pós-graduação em Ciências Biológicas, Universidade Federal de Ouro Preto, Ouro Preto, Brazil; ^4^ Departamento de Nutrição, Universidade Estácio de Sá, Rio de Janeiro, Brazil

**Keywords:** insulin resistance, oxidative stress, reactive oxygen species, inflammasome, lowgrade inflammation, phytochemicals, bioactive compounds, microRNA

## Abstract

NLRP3 inflammasome has a key role in chronic low-grade metabolic inflammation, and its excessive activation may contribute to the beginning and progression of several diseases, including hepatic insulin resistance (hIR). Thus, this review aims to highlight the role of NLRP3 inflammasome and oxidative stress in the development of hIR and evidence related to phytochemical intervention in this context. In this review, we will address the hIR pathogenesis related to reactive oxygen species (ROS) production mechanisms, involving oxidized mitochondrial DNA (ox-mtDNA) and thioredoxin interacting protein (TXNIP) induction in the NLRP3 inflammasome activation. Moreover, we discuss the inhibitory effect of bioactive compounds on the insulin signaling pathway, and the role of microRNAs (miRNAs) in the phytochemical target mechanism in ameliorating hIR. Although most of the research in the field has been focused on evaluating the inhibitory effect of phytochemicals on the NLRP3 inflammasome pathway, further investigation and clinical studies are required to provide insights into the mechanisms of action, and, thus, encourage the use of these bioactive compounds as an additional therapeutic strategy to improve hIR and correlated conditions.

## Introduction

The liver plays a key role in regulating organism homeostasis, and insulin controls hepatic metabolism mainly by regulating several proteins, such as the PI3K/AKT signaling pathway. Insulin signaling pathways, mediated by impaired tyrosine phosphorylation of insulin receptor substrates (IRS), are critical for the development of insulin resistance (IR) (Gurzov et al., 2014; Liu et al., 2016; Xu et al., 2017; Yan et al., 2018; Joseph et al., 2020; Mu et al., 2020; Abdelmageed et al., 2021) because this change in cell signaling results in detrimental effects on insulin sensitivity due to impairment on insulin-stimulated glucose uptake in hepatocytes.

The NLRP3 inflammasome has a key role in chronic low-grade metabolic inflammation, and its excessive activation may contribute to the beginning and progression of several diseases, including hepatic IR (hIR). This complex comprises a sensor (NLRP3), an adaptor [ASC (apoptosis-associated speck-like protein) or PYCARD], and an effector (caspase-1), which, upon activation, oligomerizes and activates pro-caspase-1, culminating in the release of proinflammatory cytokines IL-1β and IL-18, and pyroptotic cell death gasdermin D (GSDMD)-mediated. Numerous factors have been described as able to activate the NLRP3 inflammasome, such as inflammatory signals, proinflammatory cytokines, tumor necrosis factor (TNF) (Liu et al., 2016), glucose (Zheng et al., 2020), and oxidative stress (Kim et al., 2017; Zheng et al., 2020; Zhu et al., 2022). Thus, excessive activation may result in metabolic stress and the production of mtROS (Jia et al., 2020), affecting insulin signaling and resulting in hIR.

Because of the antioxidant and anti-inflammatory activity of phytochemicals, studies have proposed that these compounds might be candidates for use in adjunctive therapy for the treatment of several diseases involving oxidative stress and inflammation. Thus, they might interfere with inflammatory signaling pathways, involving NLRP3 inflammasome activation, mtROS, and other activating signals, and improve hIR through changes in the PI3K/AKT signaling pathway (Chen et al., 2020; Lee and Lee, 2021; Zhang et al., 2021; Kang and Chiang, 2022).

This review found that inflammasome activation is predominantly related to a high glucose diet (Zheng et al., 2020) and arsenic-induced hIR (Jia et al., 2020; Zhang et al., 2021; Qiu et al., 2022; Wu et al., 2022). Moreover, this study addressed NLRP3 inflammasome activation induced by reactive oxygen species (ROS) involving oxidized mitochondrial DNA (ox-mtDNA) and thioredoxin interacting protein (TXNIP) induction. In addition, because plants have various bioactive compounds, their extracts have been investigated to elucidate the possible mechanisms of action on metabolic syndromes, inflammation, and IR, with few or no side effects (Chen et al., 2020; Lee and Lee, 2021; Kang and Chiang, 2022; Zhang et al., 2022). However, although most of the research in the field has focused on evaluating the inhibitory effect of phytochemicals on the NLRP3 inflammasome pathway, little is known about these effects on hIR. Furthermore, the available data are limited to the effects of phenolic compounds in cells or animal models. Although the molecular mechanisms are not fully understood, most have focused on the insulin signaling pathway, and microRNAs (miRNAS) have been pointed out as a phytochemical target mechanism (Lee and Lee, 2021; Zhang et al., 2022). Thus, this review aimed to highlight the role of NLRP3 inflammasome and oxidative stress in the development of hIR and discuss evidence related to phytochemical intervention in this context.

We performed a narrative review by conducting searches and applying inclusion criteria. Specifically, we conducted an extensive search of the Pubmed, Scopus, and ScienceDirect databases, considering works published from 2012 to 2022. An internal inclusion criterion, only original articles in English were incorporated, and the search for terms was performed within the article title, abstract, and keywords. Two or three independent reviewers evaluated the studies for inclusion in the review. The keywords used are “hepatic insulin resistance” AND “oxidative stress”; “hepatic insulin resistance” AND “inflammasome”; “hepatic insulin resistance” AND “phenolic compounds”; and “hepatic insulin resistance” AND “phytochemicals.” In the final selection, after ensuring the inclusion of these terms in the title and/or abstract, we excluded duplicate papers from the databases and all articles related to nonalcoholic fatty liver disease. Furthermore, three manuscripts were discredited because they were in predatory journals. The sample was narrowed 49 original articles were selected, among them 37 *in vivo* studies, eight *in vitro* studies, three *in vitro* and *in vivo* studies, and one clinical trial study. However, to ensure a logical, complete line of reasoning, five reviews were included for further information. Thus, we reviewed 54 articles.

## Hepatic insulin resistance development and oxidative stress

### Hepatic insulin resistance development—an overview

**FIGURE 1:**
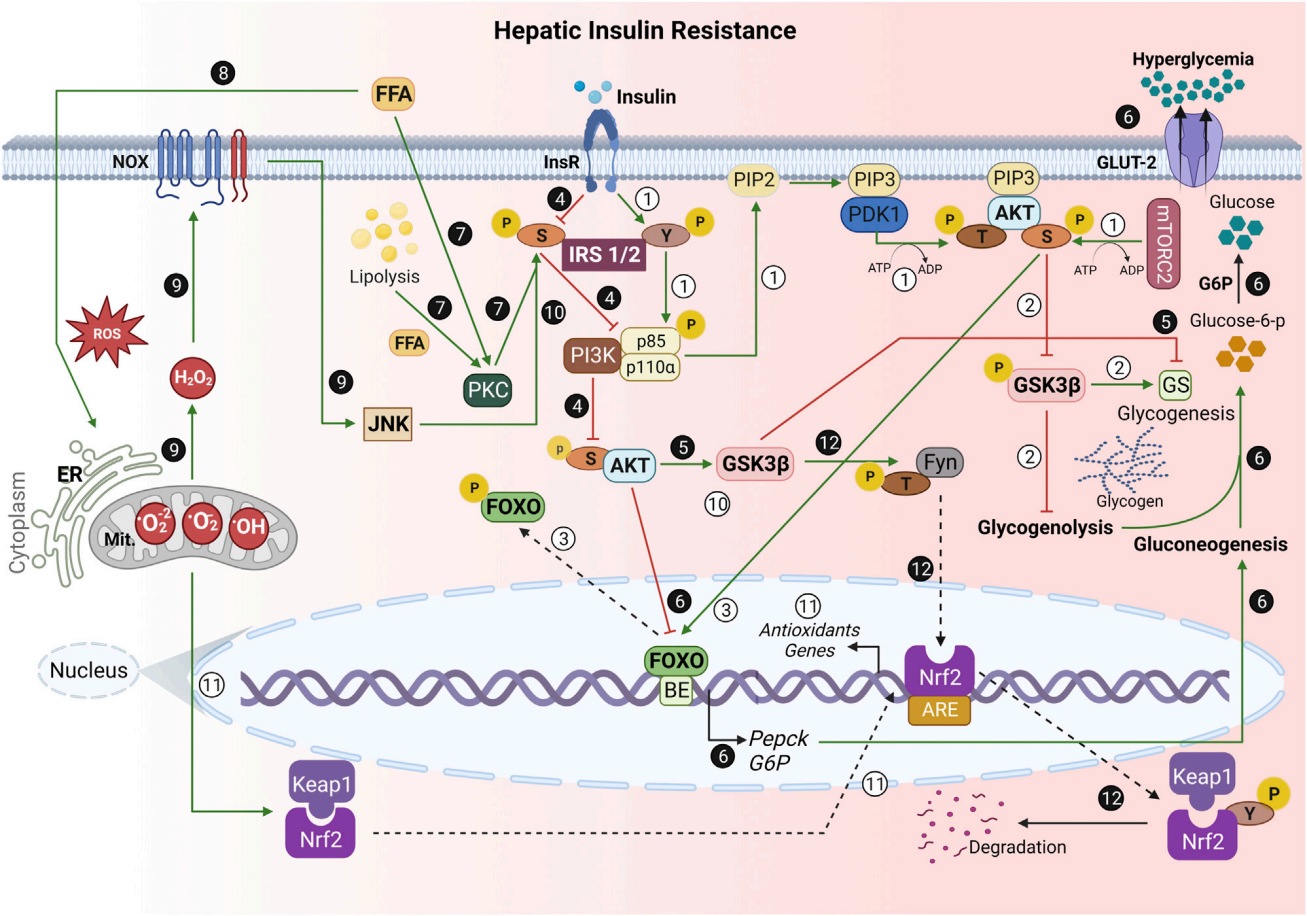
Role of oxidative stress in the hepatic insulin resistance (hIR) development. ① Under normal conditions, the binding of insulin to its receptor promotes a conformational change, which allows IRS1/2 autophosphorylation at the tyrosine (Y) residue. Then, it enables the phosphorylation of the enzyme PI3K regulatory subunit p85, reaching its catalytic domain (p110) activation. PI3K phosphorylates PIP2 at position 3 of the inositol ring, resulting in PIP3, which allows the anchoring of PDK1 and AKT. PDK1 performs the first phosphorylation of AKT at threonine residue (T) 308. mTORC2 phosphorylates AKT serine 473 [pAKT(S)]. ② pAKT(S) phosphorylates GSK3β, which inhibits glycogenolysis and activates glycogenesis through GS stimulation. ③ pAKT(S) also phosphorylates FOXO, preventing its translocation from the nucleus, culminating in PEPCK and G6P gene transcription inhibition. ❹ During IR, the S threonine kinases’ (e.g., JNK) activation phosphorylates IRS1/2 at S. ❺ Thus, there is a decrease in AKT(S) phosphorylation, allowing GSK3β activation. Therefore, there is GS inhibition. ❻ Additionally, the lack of nuclear FOXO phosphorylation by AKT(S) allows its translocation to the cytoplasm and PEPCK and G6P gene transcription. This stimulates gluconeogenesis, leading to glucose-6-phosphate production, and its dephosphorylation by G6P, which results in hyperglycemia. ❼ In addition, both FFA from the bloodstream and hepatic lipolysis activate PKC, a S/T kinase, leading to IRS1/2 phosphorylation at S, and culminate in an impairment of PI3K/AKT cascade. ❽ Mitochondrial dysfunction and ER stress due to ROS and excessive FFA are the greatest hallmarks of hIR. ❾ Moreover, the exacerbated ROS-dependent oxidative damage on hIR results in the release of H_2_O_2_ during mitochondrial respiration, contributing to increased hepatic NADPH oxidase (NOX) activity. ❿ Moreover, NOX activates JNK, which phosphorylates IRS1/2 at S307, and inhibits the physiological insulin signaling pathway. ⑪ Under homeostatic conditions, Nrf2 is sequestered in cytosol by binding to Keap1 to form a Nrf2-Keap1 complex. However, when exposed to oxidative stress, Nrf2 dissociates from Keap1, translocates into the nucleus, and activates numerous downstream antioxidants genes. 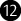
However, GSK3β activated in hIR may phosphorylate Fyn, a Y kinase translocated to the nucleus, and then phosphorylates Nrf2 at Y568. Thus, Nrf2 is nuclear exported, binds to Keap1, and is degraded. The negative regulation of Nrf2 by GSK3β/Fyn is important in repressing Nrf2 downstream genes induced in response to oxidative stress (Xu et al., 2017). IRS, insulin receptor substrate; Y, tyrosine residue; PI3K, phosphoinositide-3-kinase enzyme; PIP2, phosphatidylinositol-4,5-biphosphate; PIP3, phosphatidylinositol-3,4,5-triphosphate; PDK1, phosphoinositide-dependent kinase 1; AKT, protein kinase B (PKB); T, threonine residue; mTORC2, mammalian target protein of rapamycin complex 2; S, serine residue; GSK3, glycogen synthase kinase-3; FOXO, forkhead box; GS, glycogen synthase; PEPCK, phosphoenolpyruvate carboxykinase; G6P, glucose 6-phosphatase; JNK, c-Jun N-terminal kinase; JNK, c-Jun N-terminal kinase; FFA, free fatty acids; PKC, protein kinase C; ER, endoplasmic reticulum; ROS, reactive oxygen species; H_2_O_2_, hydrogen peroxide; NOX, nicotinamide adenine dinucleotide phosphate oxidase; Nrf2, nuclear factor erythroid 2-related factor 2; Keap1, Kelch-like ECH-associated protein; Fyn, tyrosine kinase; BE, binding elements; Mit, mitochondria; P, phosphate; GLUT, glucose transporters.→ Green arrow; upregulation/stimulation. ⊣ Red blunted arrow; downregulation/inhibition. ⇢ Black dotted arrow; translocation to/from nucleus. ↳ gene expression. Created with BioRender.com.

The liver plays a key role in regulating organism homeostasis by coordinating carbohydrate, lipid, and protein metabolism, which is fundamental to maintaining blood glucose levels by balancing gluconeogenesis and glycogenolysis. Various hormones are responsible for hepatic metabolism; however, insulin plays a central role in this regulation. After insulin binds to the InsR, the tyrosine phosphorylation of IRS results in the recruitment of phosphoinositide-3-phosphate kinase (PI3K), which catalyzes the formation of the lipid second messenger phosphatidylinositol (3,4,5)-trisphosphate (PIP3). PIP3 activates 3- phosphoinositide-dependent protein kinase (PDK1), which activates AKT/PKB and atypical protein kinase Cs (aPKCs). The PDK1 phosphorylates the activation loops of AKT/PKB on Thr308 and PKCζ on Thr410, enhancing the activity of these kinases. Next, AKT/PKB requires a second phosphorylation on Ser473, but PDK1 cannot perform that step. The existence of a PDK2 has been postulated, and evidence have revealed that the mTOR complex 2 (mTORC2) might be the PDK2 for AKT/PKB signaling (Figure 1) (Abdelmageed et al., 2021; Mata-Torres et al., 2021) [For further information see (Saltiel, 2021)].

Insulin signaling controls hepatic metabolism mainly by the regulation of several proteins, such as mammalian target protein of rapamycin (mTOR), glycogen synthase kinase 3 (GSK3), phosphoenolpyruvate carboxykinase (PEPCK), glucose 6-phosphatase (G6P), and the transcription factor forkhead box protein O (FOXO) (Gurzov et al., 2014; Joseph et al., 2020; Mu et al., 2020). The AKT activation in the liver opens up several pathways for glucose and lipid homeostasis. Once activated, this signaling controls different processes, such as gluconeogenesis, by decreasing PEPCK and G6P protein content (Abdelmageed et al., 2021) and glycogenolysis (Gurzov et al., 2014; Liu et al., 2016; Joseph et al., 2020), lipogenesis, free fatty acid (FFA) uptake, oxidation, and esterification, and the use of triacylglycerol packaging within lipoproteins for secretion by activation of sterol-regulatory element-binding protein 1c (SREBP-1), which promotes the transcription of genes involved in triglyceride (TAG) synthesis, including acetyl coenzyme A carboxylase (ACC), stearoyl coenzyme A desaturase (SCD-1) and fatty acid synthase (FAS) (Keenan et al., 2019). Whichever PI3K/AKT signaling deregulation culminates in reduced glucose utilization and glycogenesis, hepatic glucose output, glycogenolysis, and triglyceride accumulation are increased (Gurzov et al., 2014).

Therefore, many factors contribute to hIR development. Oxidative stress, inflammation, and excessive FFA are also observed in the progression of hIR because mitochondrial dysfunction-induced inflammation and endoplasmic reticulum (ER) stress are the greatest hallmarks of hIR (Pereira et al., 2014; Pereira et al., 2015). All these factors promote hIR by activating PKCδ, c-Jun-N-terminal kinase (JNK), kappa-B kinase nuclear factor (NF-κB), kappa-B kinase inhibitor (IKKβ), and nicotinamide adenine dinucleotide phosphate oxidase (NADPH oxidase) (Pereira et al., 2014). Notably, PKC increases serine/threonine residues phosphorylation of IRS and impairs subsequent activation of the downstream PI3K/AKT cascade, worsening the hIR in the presence of FFA (Figure 1). In addition, the accumulation of FFA in the liver has a distinct role in IR development. Pereira et al. (2014), using a 7 h infusion of intralipid + heparin (IH) to elevate circulating FFA in Wistar rats, observed that PKCδ and oxidative stress played causal roles in hIR (Pereira et al., 2014). He et al. (2020) also demonstrated that increased plasma concentrations of serum nonesterified fatty acid (NEFA) and glycerol promoted an increase in hepatic gluconeogenesis via the glycerol-dihydroxyacetone phosphate and increased hepatic lipid accumulation, which favors the development of IR (He et al., 2020).

Moreover, the impairment in the hepatic insulin signaling culminates in gluconeogenesis increases, which contributes to hyperglycemia, and induces hepatic lipid synthesis, exacerbating steatosis and hypertriglyceridemia (Shlomai et al., 2012; Gurzov et al., 2014; Fang et al., 2020; Joseph et al., 2020). This process induces a vicious cycle in glucose and lipid metabolism: the unsuppressed gluconeogenesis results in hyperglycemia, which might be used as a substrate for the hepatic lipogenesis, further aggravating the hIR (Gurzov et al., 2014; Dong et al., 2015; Liu et al., 2016; Joseph et al., 2020).

Furthermore, there is adipocyte lipolysis, which increases FFA flow to the liver, culminating with hepatic fat accumulation (Keenan et al., 2019). Altogether, these events play a central role in the pathogenesis of the systemic IR (Wang et al., 2013). In addition, excessive oxidative metabolism activates inflammatory pathways, which impair insulin action (Satapati et al., 2012). Moreover, research has suggested that oxidative stress and inflammation are associated with hIR development (Liu C.-M. et al., 2017; Xu et al., 2017; Joseph et al., 2020), discussed further in the next section.

### Role of oxidative stress in development of hepatic insulin resistance–from 2012 to 2022

Oxidative stress has been linked to hIR and is a critical factor in its development (Wu et al., 2013; Kahle et al., 2015; Kucejova et al., 2016; Liu et al., 2016; Valencia et al., 2016; Liu P. et al., 2017; Xu et al., 2017; Yan et al., 2018; Joseph et al., 2020; Abdelmageed et al., 2021; Altamura et al., 2021). However, although this phenomenon has been extensively studied, the underlying molecular mechanisms remain poorly understood. Oxidative stress is an imbalance between oxidation and antioxidant defense, which can lead to the generation of free radicals (Liu C.-M. et al., 2017). In the liver, the increase in ROS production due to mitochondrial dysfunction and ER stress enhances lipid peroxidation, leading to the formation of aldehyde by-products, such as malondialdehyde (MDA) (Satapati et al., 2012). Moreover, ER stress disturbs the protein-folding processes triggering the unfolded protein response (UPR), which results in proteotoxicity that contributes to IR development and apoptosis (Lee and Lee, 2021). The interaction between oxidative stress and hIR can accelerate cellular injury and inflammation and might result in liver fibrosis. In addition, the imbalance in lipid homeostasis results in the generation of toxic lipids (i.e., ceramides), which may activate proinflammatory cytokines and inhibit the PI3K/AKT pathway. Furthermore, the continued increases in mitochondrial ROS (mtROS) generation exacerbated by FFA oxidation activates serine-threonine kinase cascades, leading to decreases in tyrosine IRS phosphorylation, and impairment in insulin signaling (Figure 1) (Ramirez et al., 2013). To reinforce this concept, Crescenzo et al. (2018) demonstrated that fructose-induced hIR results in increases in ceramides in young rats (aged 30 days) and decreases in Tyr-IRS1 and AKT phosphorylation, increasing the hepatic nitrotyrosine levels (Crescenzo et al., 2018).

The mtROS overload results in activating various stress-sensitive intracellular signal transduction pathways. As previously described in the literature, excessive mtROS production can reduce Tyr phosphorylation of IRS-1/2 and induce Ser phosphorylation of IRS-1/2 by activating JNK and NF-κB, an inflammatory signal involved in IR through the serine phosphorylation of IRS1. This induces IR owing to a decrease in the insulin sensitivity of hepatocytes, because there are decreases in PI3K/AKT cascade (Figure 1) (Liu P. et al., 2017; Yan et al., 2018; Mu et al., 2020).

NF-κB is a primary transcription factor in inflammatory diseases and may influence hIR progression. The signaling pathway comprises NF-κB activation and inactivation, wherein inactivated NF-κB in the cytosol binds to the inhibitor of κB (IκB) molecules and prevents its transcriptional function. Conversely, IKK is activated by extracellular stimuli (e.g., ROS) and phosphorylates IκB, which promotes the NF-κB detachment from the IKK complex and results in its nuclear translocation. Thus, the NF-κB translocation leads to increases in inflammatory cytokines production (e.g., IL-6, TNF, and IL-1β), which have a pivotal role in hIR development (Figure 1) (Liu et al., 2016). Zheng et al. (2018) observed that sitagliptin treatment (50 mg/kg d/4 weeks) using a dipeptidyl-peptidase 4 inhibitor reduced hIR in ob/ob mice. The sitagliptin-treated ob/ob mice showed a decrease in NF-κB p65 activity and JNK phosphorylation, culminating in the downregulation of TNF and IL-6 expression. Moreover, sitagliptin increased the AKT and AMPK phosphorylation levels and decreased those of mTOR. Thus, sitagliptin might ameliorate hIR development in ob/ob mice, inhibiting inflammatory responses via the AMPK/mTOR signaling pathway (Zheng et al., 2018).

Moreover, the increase in ROS-dependent oxidative damage on hIR results in an increase in the release of hydrogen peroxide during mitochondrial respiration, contributing to increased hepatic NADPH oxidase (NOX) activity. NADPH oxidase is a main regulator for H_2_O_2_ production (Figure 1), and its activity is controlled by mitogen-activated protein kinases (MAPKs), such as ERK1/2 (extracellular signal-regulated kinase 1/2), p38 mitogen-activated protein kinase (p38MAPK), and JNK (Pereira et al., 2014).

Researchers have shown that oxidative stress is one of the stressors that activate the JNK signaling that plays a crucial role in hIR progression. Therefore, activated JNK phosphorylates IRS 1/2 at Ser307, reducing the AKT phosphorylation at Ser473 and thus inhibiting the physiological insulin signaling pathway (Figure 1) (Cao et al., 2014; Tang et al., 2015; Liu P. et al., 2017; Xu et al., 2017; Yan et al., 2018; Mu et al., 2020). For elucidating the role of ROS-mediated JNK pathway signaling in hIR development, Zhou et al. (2015) observed that high-dose selenite treatment impaired hepatic IRS1/AKT/FOXO1 signaling, upregulating the PEPCK, G6P, PGC-1α gene expressions and increasing MDA and protein carbonyl contents, with a decrease in glutathione (GSH)/glutathione oxidized (GSSG) ratio in the liver. They also found an increase in the apoptosis signal-regulated kinase 1 (ASK1)/MAPK kinase 4 (MKK4)/JNK signaling pathway, suggesting that high-dose selenite treatment exacerbates hIR, at least in part, through the oxidative stress-mediated JNK pathway (Zhou et al., 2015). Additionally, Liu P. et al. (2017) demonstrated that the C-terminal-binding protein 2 (CtBP2) over-expressed in HepG2 cells might ameliorate palmitate (PA)-induced IR due to the inhibition of the ROS-dependent JNK pathway. They observed an increase in GSK3β and AKT phosphorylation, suggesting the critical role of CtBP2 in hIR development. Furthermore, the over-expressed CtBP2 reversed the effects of PA on ROS formation, lipid accumulation, and gluconeogenesis, improving insulin sensitivity (Liu P. et al., 2017).

After demonstrating that oxidative stress plays a crucial role in the development of hIR, some works have demonstrated the role of antioxidants as a strategy to attenuate hIR progression. Furthermore, Pereira et al. (2014) demonstrated that N-acetyl-l-cysteine (NAC), an antioxidant, prevented IH (intralipid + heparin)-induced hIR, avoiding the IRS 1/2 phosphorylation at Ser307 by JNK, improving hepatic insulin signaling. Moreover, Pereira et al. (2014) have shown that an antisense oligonucleotide against PKCδ prevented hIR by decreasing the phosphorylation of p47phox (marker of NOX activation) in IH infusion. Similar to NAC, apocynin, an o-methyl catechol characterized as an NOX inhibitor, also prevented IH-induced hIR (Pereira et al., 2014). Notably, the nuclear factor erythroid 2-related factor 2 (Nrf2) pathway has a critical role in antioxidative stress due to the antioxidant genes and detoxification enzymes expression regulation (Dong et al., 2015; Liu et al., 2016; Liu P. et al., 2017; Xu et al., 2017). Nrf2 pathway activation or induced expression regulates the antioxidant response element (ARE)-mediated expression of detoxifying and antioxidant enzymes that protect against the adverse effects of oxidative stress induced by ROS. The Nrf2 pathway has significantly improved glucose homeostasis and insulin sensitivity *in vivo* and *in vitro,* respectively (Dong et al., 2015; Liu et al., 2016; Liu P. et al., 2017; Xu et al., 2017). Under homeostatic conditions, Nrf2 is sequestered in the cytosol by binding to Kelch-like ECH-associated protein (Keap1) to form an Nrf2-Keap1 complex. However, when exposed to oxidative stress, Nrf2 dissociates with Keap1, translocates into the nucleus, and activates numerous downstream genes (Figure 1) (Dong et al., 2015; Liu et al., 2016; Liu P. et al., 2017; Xu et al., 2017). Remarkably, the Nrf2 and NF-κB pathways regulate the physiological homeostasis of cellular redox status and responses to stress and inflammation. Studies have proposed that Nrf2 plays a critical role in counteracting the NF-κB driven inflammatory response in various experimental models. For example, Liu et al. (2016) demonstrated that Nrf2-null mice HFD-treated for 8 weeks had decreased IRS1 Tyr phosphorylation in the liver. They also observed that Nrf2-null mice had NF-κB activation, and that cytokine IL-6 and TNF expression increased, with an increase in hepatic MDA and a decrease in glutathione levels when fed an HFD, suggesting that the consequential hIR observed might be due to NF-κB pathway activation owing to oxidative stress (Liu et al., 2016).

The literature has demonstrated that the absence of phosphorylation of GSK3β by AKT regulates downstream Nrf2 activation-induced gene expression. Non-phosphorylated GSK3β activates cytosolic Fyn, a tyrosine kinase that is translocated to the nucleus, and then, phosphorylates Tyr 568 of Nrf2. This results in the Nrf2 nuclear exportation, which binds to Keap1, and it is degraded (Figure 1). The negative regulation of Nrf2 by GSK3β/Fyn is important in repressing Nrf2 downstream genes that were induced in response to oxidative stress (Xu et al., 2017).

As we have discussed, one mechanism that may link metabolic stress and hIR pathogenesis is chronic low-grade metabolic inflammation (metaflammation) (Zheng et al., 2020). In this manner, the NLRP3 inflammasome has a crucial role in metaflammation development, and there is evidence that relates the excessive NLRP3 inflammasome activation to the beginning and progression of several diseases, such as hIR. Furthermore, reports have regarded NLRP3 inflammasome activation and insulin sensitivity in diabetic db/db mice livers (Kim et al., 2017) and HFD-exposed mice hepatocytes (Zheng et al., 2020). Therefore, NLRP3 inflammasome activation and their correlated pathways, involving mtROS and other activating signals, are related to hIR progression and are discussed as follows.

## Role of NLRP3 inflammasome and oxidative stress in the development of hepatic insulin resistance

### NLRP3 inflammasome: a short overview

The NLRP3 inflammasome (which encompasses NOD-, LRR- and pyrin domain-containing protein 3) is a multiprotein complex that plays a crucial role in regulating the innate immune system and inflammatory signaling. This complex is made up of a sensor (NLRP3), an adaptor [ASC (apoptosis-associated speck-like protein) or PYCARD], and an effector (caspase-1), which, upon activation, oligomerizes and activates pro-caspase-1, culminating in the release of proinflammatory cytokines IL-1β and IL-18, and pyroptotic cell death gasdermin D (GSDMD)-mediated (Kim et al., 2017; Islam et al., 2020; Jia et al., 2020; Zheng et al., 2020; Zhang et al., 2021; Qiu et al., 2022; Wu et al., 2022) [For further information, please see in (Swanson et al., 2019)].

**FIGURE 2:**
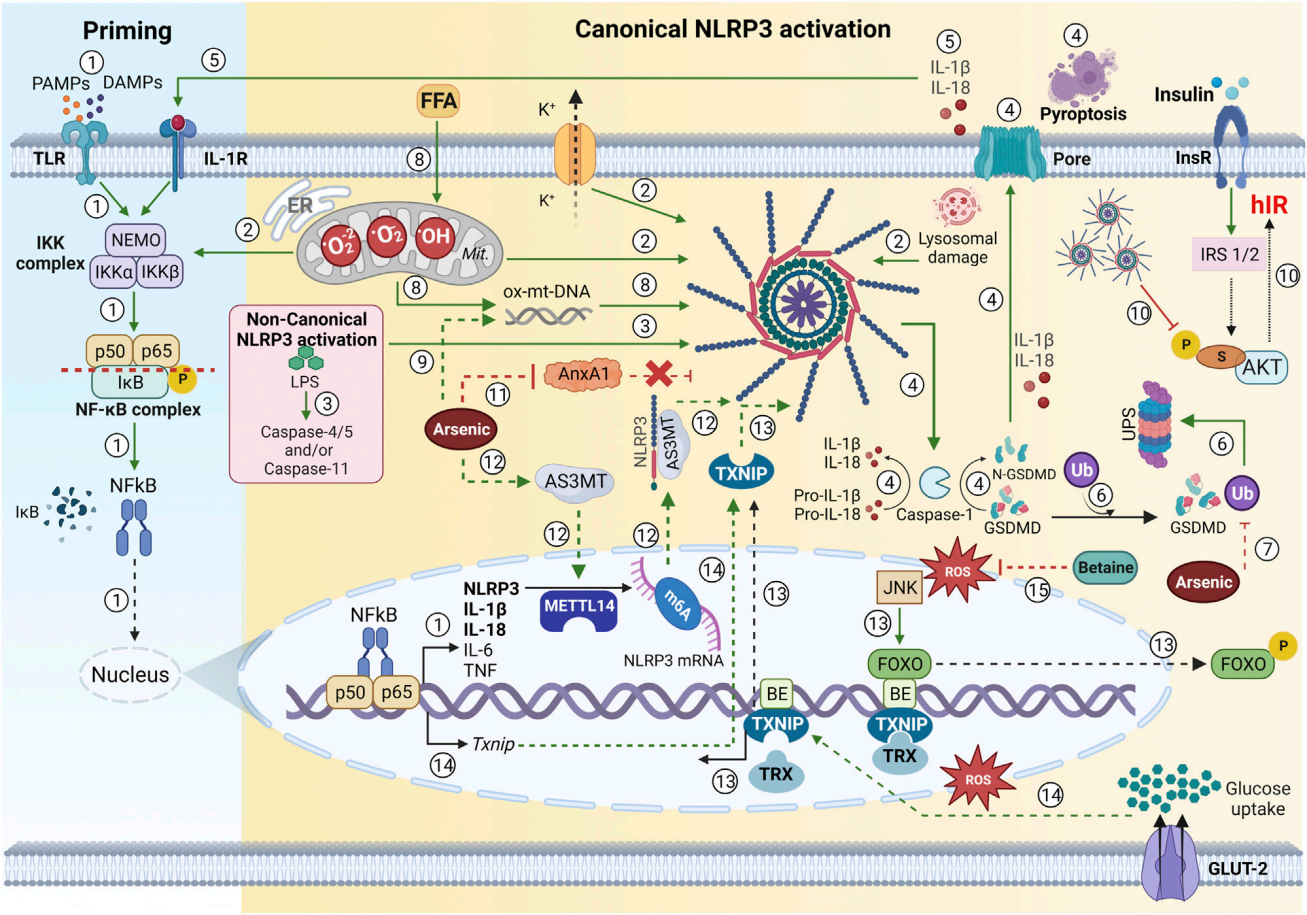
Oxidative stress in NLRP3 inflammasome priming—an overview. ① Early recognition of PAMPS and DAMPS in the canonical inflammasome pathway to TLR activates the NF-κB pathway and triggers the recruitment and activation of the IKK complex (which includes IKKα, IKKβ, and two molecules of non-enzymatic regulatory protein -NEMO). This complex activates NF-kB dimers (p50 and p65) through IKβ subunit phosphorylation, which is then degraded. Thus, NF-kB is translocated into the nucleus and activates inflammasome gene expression (NLRP3, pro-IL-1β, pro-IL-18, pro IL-6, and TNF). ② An activating signal due to the disruption in cellular physiology (mtROS, lysosomal damage, or cytosolic K+ efflux) triggers the NLRP3 inflammasome assembly. In addition, ROS has been proposed to activate the IKK complex. ③ LPS is also able to activate the non-canonical NLRP3 pathway, due to caspase-4 or caspase-5 (human), and mouse caspase-11, which indirectly promotes the pro-IL-1β or pro-IL-18 cleavage. ④ The NLRP3 inflammasome assembly results in caspase-1 activation, culminating in pro-IL-1β and pro-IL-18 cleavage and release. Caspase-1 also cleaves to the GSDMD, which results in the release and insertion of its cytotoxic N-terminal portion in the membrane, promoting pore formation and inducing pyroptosis. ⑤ Both mature interleukins may be recognized by IL-1R and trigger the NF-κB signaling pathway, generating a continuous positive upregulation in the inflammatory response. ⑥ GSDMD ubiquitination led to a reduction in the release of IL-1β and IL-18, improving glucose metabolism. ⑦ Notably, GSDMD ubiquitination is inhibited in arsenic-induced IR, with its intracellular degradation reduced, resulting in GSDMD accumulation, pyroptosis, and hIR (Zhu et al., 2022). ⑧ Both FFA from hepatic lipolysis and circulation may be oxidized excessively and generate mtROS. Excessive mtROS production results in mitochondria dysfunction and ox-mtDNA releases, which can also activate the NLRP3 inflammasome and change glucose metabolism. ⑨ Arsenic-exposed hepatocytes increased altered mitophagy, leading to the generation of mtROS and ox-mtDNA, which culminates in an increase in IL-1β and IL-18 and glucose metabolism disorder (Jia et al. (2020). ⑩ NLRP3 inflammasome activation worsens insulin sensitivity on hepatocytes due to a decrease in p-AKT (Ser307) phosphorylation, which impairs the IRS/AKT signaling and results in hIR. ⑪ Arsenic-induced hIR inhibits the anti-inflammatory factor Annexin A1 (AnxA1), which was shown to be a promising therapeutic target on hIR due to its ability to bind to NLRP3 inflammasome and avoid its activation (Wu et al., 2022). ⑫ Arsenic promotes the upregulation of AS3MT, which activates METTL14 and stabilizes m6A-modified *Nlrp3* mRNA, suggesting that m6A modification critically regulates NLRP3 inflammasome activation and contributes to arsenic-induced hIR onset (Qiu et al., 2022). ⑬ During hIR, excessive mtROS levels may activate the JNK pathway and phosphorylates FOXO, which is nuclear exported and allows *Txnip* expression, which culminates in TXNIP/NLRP3 axis inflammasome activation. ⑭ Moreover, high glucose concentrations led to the dissociation of TXNIP from TRX, and the increased ROS-induced Txnip gene expression, triggering the TXNIP/NLRP3 axis promoting inflammasome activation. Furthermore, TXNIP expression is NF-κB-induced and contributes to NLRP3 inflammasome oligomerization (Zheng et al., 2020). ⑮ Betaine (from *Lycium chinense*) water extracts inhibited FOXO1 activation and prevented IL-1β production through the inhibition of NLRP3 inflammasome activation via FOXO1 and TXNIP interaction in hepatic cells (Kim et al., 2017). PAMPs, pathogen-associated molecular patterns; DAMPs, endogenous danger-associated molecular patterns; TLR, Toll-like receptor family; NF-κB, nuclear factor kappa beta; IKK, IkappaB kinase; NEMO, NF-κB essential modifier; IK, IkappaB; NLRP3, Nod like receptor family pyrin domain-containing 3; NLRP3, Nod like receptor family pyrin domain-containing 3; NLRP3, Nod like receptor family pyrin domain-containing 3; IL, interleukin; TNF, tumor necrosis factor; mtROs, mitochondrial reactive oxygen species; K, potassium; ROS, reactive oxygen species; LPS, lipopolysaccharides; GSDMD, gasdermin D; IL-1R, interleukin 1- receptor; IR, insulin resistance; hIR, hepatic insulin resistance; FFA, free fatty acids; ox-mtDNA, oxidized mitochondrial DNA; AKT, protein kinase B (PKB); AnxA1, Annexin A1; AS3MT, arsenite methyltransferase; METTL14, methyltransferase-like 14; m6A, N6-methyladenosine; JNK, Jun N-terminal kinase; FOXO1, forkhead box 1; TXNIP, thioredoxin interacting protein; Mit, mitochondria; ER, endoplasmic reticulum; TRX, thioredoxin; P, phosphate; BE, binding elements. → Green arrow; upregulation/stimulation. ⊣ Red blunted arrow; downregulation/inhibition. ⇢ Black dotted arrow; translocation to/from nucleus. ↳ gene expression. Green or red dotted arrow; possible mechanisms described in the literature. Created with BioRender.com.

The NLRP3 inflammasome can be activated by the canonical and noncanonical signaling pathway (Jia et al., 2020). In the canonical pathway, early recognition of pathogen-associated molecular patterns (PAMPs) and endogenous damage-associated molecular patterns (DAMPs), such as glucose (Kim et al., 2017; Jia et al., 2020; Zheng et al., 2020), cholesterol crystals, uric acid (Kim et al., 2017; Jia et al., 2020; Zheng et al., 2020), palmitate, C-reactive protein (CRP), ceramide, islet amyloid polypeptide (Zheng et al., 2020) and saturated fatty acids (Zhang et al., 2021), activate caspase-1 directly, inducing IL-1β and IL-18 cleavage and secretion and pyroptosis GSDMD-mediated (Figure 2) (Kim et al., 2017; Zheng et al., 2020; Wu et al., 2022). The noncanonical inflammasome pathway is activated by intracellular lipopolysaccharide (LPS); this pathway is mostly dependent on human caspase-4 and caspase-5 or mouse caspase-11, which indirectly promotes the production of pro-IL-1β or pro-IL-18 (Figure 2) (Jia et al., 2020).

NLRP3 inflammasome activation occurs in two steps: 1- a priming signal [PAMPs and DAMPs in the canonical or intracellular lipopolysaccharide (LPS) in the noncanonical inflammasome pathway] that activates the NF-κB pathway via the stimulation of proinflammatory receptors, such as the Toll-Like receptor family (TLRs), and interleukin receptors (IL-1R), which induces the expression of NLRP3, pro-IL-1β, and pro-IL-18 (Jia et al., 2020), and 2- an activating signal due to the disruption in cellular physiology (glucose, mtROS, lysosomal damage, or cytosolic K+ efflux), which triggers the NLRP3 inflammasome assembly and caspase-1 activation, culminating in pro-IL-1β and pro-IL-18 cleavage and release (Figure 2) (Kim et al., 2017; Jia et al., 2020; Zheng et al., 2020; Wu et al., 2022). Both mature interleukins are recognized by the IL-1 receptors and trigger the NF-κB signaling pathway, generating a continuous positive upregulation in inflammatory response, such as a positive feedback mechanism (Zheng et al., 2020). In parallel with the increase in IL-1β and IL-18 expression by NF-κB, the NLRP3 inflammasome activation results in the GSDMD cleavage by caspase-1. This result in the release and insertion of its cytotoxic N-terminal portion in the membrane, promoting pore formation and inducing pyroptosis (Figure 2) (Zhu et al., 2022). That inflammation mediated by NLRP3 inflammasome plays a crucial role in the development of IR. The mechanism behind this phenomenon involves a block in IRS signaling (IRS/AKT) by NLRP3 inflammasome activation, and, thus, suppresses cellular insulin-stimulated glucose uptake in hepatocytes (Jia et al., 2020). Moreover, the GSDMD expression and cleavage by caspase-1 is related to hIR development because the GSDMD inhibition led to a reduction in the release of IL-1β and IL- 18, improving glucose metabolism (Zhu et al., 2022). Notably, GSDMD ubiquitination is inhibited in arsenic-induced IR, with its intracellular degradation reduced, resulting in GSDMD accumulation, pyroptosis, and hIR. Zhu et al. (2022) demonstrated that GSDMD might be ubiquitinated by K48- and K63-linked ubiquitination and degraded by the ubiquitin-proteasome system and autophagy-lysosome pathway, two key protein degradation systems in eukaryotic cells that help protein and organelle homeostasis. Thus, the exploration of GSDMD ubiquitination modification and degradation provided a paradigm to investigate the relationship between pyroptosis signals and disease, contributing to understanding these mechanisms and proposing hIR new treatment perspectives (Figure 2) (Zhu et al., 2022).

### NLRP3 inflammasome activation and hepatic insulin resistance development—role of oxidative stress

Regarding the insulin signaling pathway, IRS-1 is one of the substrates for tyrosine kinase activity from InsR, and its phosphorylation at serine residues typified by Ser307 can mediate IR development. The next step is a decrease in insulin-induced phosphorylation of AKT at Ser473 [p-AKT (Ser473)], negatively affecting insulin sensitivity due to impaired insulin-stimulated glucose uptake in hepatocytes (Figure 2) (Jia et al., 2020; Zhang et al., 2021).

According to the literature, a high glucose diet (Zheng et al., 2020) and arsenic-induced IR (Jia et al., 2020; Zhang et al., 2021) lead to NLRP3 inflammasome activation. They worsen the insulin sensitivity of hepatocytes owing to a decrease in p-AKT (Ser307) phosphorylation, impairing IRS/AKT signaling (Figure 2) (Jia et al., 2020; Zheng et al., 2020). The presence of Kupffer cells in hepatocytes exposed to high glucose or arsenic may exacerbate the decrease in insulin-induced AKT phosphorylation and increase GSK3β, suggesting that progress in liver inflammation would result in hepatic insulin sensitivity impairment. This hypothesis is supported by the increase in levels of NLRP3, pro-caspase-1, caspase-1, pro- IL-1β, and IL-1β in hepatocytes lysates and IL-1β in cell culture supernatants, corroborating the idea that harm to hepatic insulin sensitivity may be due to an advance in liver inflammation in murine models (Zheng et al., 2020).

Regarding NLRP3 inflammasome activation and consequently hIR, Wu et al. (2022) demonstrated that arsenic-induced hIR inhibited Annexin A1 (AnxA1) activity, an anti-inflammatory factor that had its mRNA and protein levels AnxA1 downregulated in rat livers and L-02 cells, suggesting that the exogenous supplementation of AnxA may be a promising therapeutic target for hIR. Likewise, they showed that AnxA1 overexpression inhibited NLRP3 inflammasome activation by binding to NLRP3, preventing the development of hIR (Figure 2) (Wu et al., 2022).

RNA methylation has emerged as an active research field because N6-methyladenosine (m6A) mRNA methylation-mediated post-transcriptional modification is involved in many biological processes, including metabolism (Zhang et al., 2022). The evidence shows that the abnormal levels of m6A and key methylesterase of m6A are related to IR, inflammatory, oxidative stress, and other mechanisms (Zheng et al., 2020; Qiu et al., 2022; Zhang et al., 2022); methyltransferase-like 14 (METTL14)-mediated m6A methylation enhanced *Nlrp3* mRNA stability; and arsenite methyltransferase (AS3MT) interacted with NLRP3 to activate the inflammasome (Figure 2), contributing to the onset of arsenic-induced hIR. Moreover, AS3MT strengthened the association between the m6A methylase and NLRP3, stabilizing m6A-modified NLRP3, suggesting that m6A modification critically regulates NLRP3 inflammasome activation during arsenic-induced hIR, opening new directions for therapeutic interventions in IR context (Qiu et al., 2022).

According to the previous statement, NLRP3 inflammasome activation is a two-step process: a priming signal (expression of NLRP3, pro-IL-1β, and pro-IL-18) and an activating signal, such as ROS, which triggers the NLRP3 inflammasome assembly. All known NLRP3 activators generate ROS, inducing a specific protein conformational change that subsequently activates the NLRP3 inflammasome. In this review, we address the ROS production mechanisms involving ox-mtDNA and TXNIP induction (Figure 2) (Kim et al., 2017; Jia et al., 2020; Zheng et al., 2020).

Mitochondrial dysfunction is closely related to IR pathogenesis, due to mtROS production, which contributes to NLRP3 inflammasome activation. Excessive mtROS production impairs mitochondria, and they release ox-mtDNA, which can also activate the NLRP3 inflammasome (Jia et al., 2020). The cellular process that removes impaired mitochondria is called mitophagy, and any altered removal of damaged mitochondria may contribute to the activation of the NLRP3 inflammasome. Jia et al. (2020) demonstrated that arsenic-exposed hepatocytes increased altered mitophagy, leading to the generation of mtROS and ox-mtDNA, which culminates in IL-1β, and IL-18 increases, and a glucose metabolism disorder occurs (Figure 2). They demonstrated that the use of a specific scavenger for mtROS (named TEMPO) resulted in the downregulation of mitophagy and ox-mtDNA, inhibited NLRP3 inflammasome activation, and the basal protein expression of NLRP3 and IL-1β in in arsenic-exposed hepatocytes (Jia et al., 2020).

Another mechanism involving the relationship between ROS and NLRP3 inflammasome activation is related to TXNIP. Inflammasome activators induce the dissociation of TXNIP from TRX in a ROS-sensitive manner, allowing it to bind to NLRP3. Zheng et al. (2020) demonstrated that high glucose concentrations led to the dissociation of TXNIP from TRX, and the increased *Txnip* gene expression was ROS induced, triggering the TXNIP/NLRP3 axis and thus promoting inflammasome activation. Furthermore, TXNIP expression is NF-κB-induced, and it also contributes to NLRP3 inflammasome oligomerization (Figure 2) (Zheng et al., 2020). The involvement of TXNIP in NLRP3 inflammasome activation may provide the link between IL-1 and IR. TXNIP is a target of transcription factor FOXO1, which was reported to bind to the TXNIP promoter (Figure 2) *in vivo* in human islets, INS-1 β, and liver, downregulating TXNIP expression (Kim et al., 2017). FOXO factor phosphorylation by insulin-induced AKT results in its translocation from the nucleus to the cytoplasm (Kim et al., 2017).

During hIR, there is a decrease in insulin-induced phosphorylation of AKT at Ser473, which would decrease FOXO factor phosphorylation and would prevent its translocation from the nucleus to the cytoplasm. However, the elevated ROS levels during hIR may activate FOXO factors via the JNK pathway, resulting in its translocation to the cytoplasm and allowing Txnip expression, resulting in TXNIP/NLRP3 axis inflammasome activation (Kim et al., 2017). In this regard, Kim et al. (2017) proposed the attenuation of the TXNIP/NLRP3 axis inflammasome activation through the administration of betaine (from *Lycium chinense*) water extracts to the db/db mice via oral gavage (50 mg/kg/day) for 3 weeks. They found downregulation in NLRP3 inflammasome genes, decreasing ROS production in hepatic cells. Additionally, betaine inhibited insulin-induced PI3K/AKT signaling and FOXO1 activation, and their results have suggested that betaine prevented the IL-1β production through the inhibition of NLRP3 inflammasome activation via FOXO1 and TXNIP interaction (Figure 2) (Kim et al., 2017).

Based on the description in the literature of the relationship between NLRP3 inflammasome activation and hIR development, questions have been raised regarding new perspectives for preventing and treating hIR. In this line of thinking, various phytochemicals have been proposed as anti-inflammatory agents for complementary or alternative anti-inflammatory therapeutics in the treatment of chronic inflammatory disorders, such as hIR; some of them are discussed in the next section.

### Role of phytochemicals ameliorating NLRP3 inflammasome activation and oxidative stress: potential mechanisms involved in improving hepatic insulin resistance

The potential of phytochemicals for treating several diseases involving oxidative stress and inflammation has received considerable attention. Phytochemicals are secondary metabolites naturally present in plants. The most predominant classes of phytochemicals are polyphenols, terpenoids, alkaloids, carotenoids, phytosterols, saponins, glucosinolates, and polysaccharides. These compounds may have bioactive properties, such as antioxidant, anti-inflammatory, anti-cancer properties, and are a safe, low-cost, and efficient alternative to treat several chronic diseases. Thus, they probably interfere with inflammatory signaling pathways, involving NLRP3 inflammasome activation, mtROS, and other activating signals, and improve hIR (Chen et al., 2020; Lee and Lee, 2021; Zhang et al., 2021; Kang and Chiang, 2022).

The NF-κB pathway is commonly cited as being inhibited by phytochemicals (e.g., flavonoids, phenols, and terpenes). Studies of flavonoids (e.g., cardamonin, quercetin, luteolin, and apigenin) have described that the action of these bioactive compounds on inflammation is mediated by the NLRP3 inflammasome through different mechanisms, and many of mechanisms are non-selective. Notably, most flavonoids (e.g., cardamonin, luteolin, isoliquiritigenin, and glycyrrhizin) can act on the Nrf2-mediated antioxidant signaling pathway, decreasing ROS production, and negatively regulate NLRP3 activation. Notably, quercetin and apigenin have an inhibitory action on MAPK and janus kinase/signal transducers and activators of transcription (JAK/STAT) pathways, and apigenin also acts by preventing the formation of ASC speck. All these compounds can act on priming and/or activation steps, inhibiting the NLRP3 inflammasome pathway. Phenolic compounds (e.g., artemisia, curcumin, caffeic acid phenethyl ester, and obovatol) have anti-inflammatory and antioxidant effects that suppress inflammatory mediators. These compounds act selectively on NLRP3 inflammasome activation, interfering with ASC speck formation, caspase-1 cleavage, and IL-1β secretion or blocking ROS production, and inhibit NF-κB, JNK, and ERK pathways. Regarding terpenoid members (e.g., oridonin, parthenolide, and andrographolide), they can inhibit NF-κB signaling, interfering with its translocation to the nucleus or suppressing the NF-κB and MAPK pathways. Moreover, terpenoids may bind to NLRP3 and disrupt NLRP3-NIMA-related kinase 7 (NEK7) interactions, which are critical for inflammasome activation and assembly. Finally, sulforaphane and shikonin can activate the Nrf2 transcription factor and act as a non-selective inhibitor of inflammasome pathways, inhibiting NLRP3-mediated IL-1β secretion and pro-caspase-1 production and cleavage through NF-κB suppression activation [For further information, see (Blevins et al., 2022)].

Although most of the scientific research on phytochemicals has evaluated the inhibitory effect on the NLRP3 inflammasome pathway, few studies have deepened the knowledge on these effects on hIR. Furthermore, the available data on hIR are from studies conducted with cells or animals and include other metabolites able to act as bioactive compounds. Although the molecular mechanisms are not fully understood, most of research has focused on the insulin signaling pathway in the liver, exploring alterations in PI3K/AKT signaling, and miRNAS are highlighted as a promising phytochemical target mechanism (Lee and Lee, 2021; Zhang et al., 2022). Furthermore, most phytochemicals act as ROS scavengers on inflammatory pathways implicated in IR pathology. Table 1 lists the bioactive compounds involved in improving hIR addressed in this review.

**TABLE 1:** Bioactive compounds involved in improving hepatic insulin resistance.

Class	Compound	Outcomes	Research model	Reference
Phenolics	SOL	- Positive effect on glucose homeostasis: ↓AMPK/AKT/GSK3β pathways; ↓GSK3β activated	HepG2 cells (*in vitro*) Sprague-Dawley rats	Chen et al. (2020)
	AMBE	- Improve glucose tolerance: ↓glycemia, insulinemia, and HOMA-IR index; ↑glucokinase and pyruvate kinase enzymes activities, ↑hepatic glycogen in T2DM rats; ↓PEPCK and G6P activities, ↓hepatic gluconeogenesis;- Improve oxidative stress and inflammation: ↓ROS production and MDA levels, ↑GSH activity; ↓ *Il-1b, Il-6* hepatic mRNA; ↓TNF expression; - hIR attenuation through PI3K/AKT signaling pathway: ↑p-IRS-2, p-PI3K, p-Akt, p-GSK3β, and GLUT2	Male Wistar rats	Mu et al. (2020)
	RSV + FA + EGCG	- Improve glucose metabolism regulation: ↑GLUT4 translocation in IR muscle cells, regulating both insulin-independent (calcium and AMPK) and insulin-dependent (PI3K) signaling molecules; ↑glycogen synthesis and ↓glucose production by IR liver cells.- Improve carbohydrate and lipid metabolism, ameliorating the muscle and hIR	Rat myoblast-like cells (*in vitro*) Male C57BL/6 mice	Kang and Chiang (2022)
Phenolic acids	PCA	- Improve glucose tolerance and homeostasis: ↓fasting glycemia and insulinemia, ↓ HOMA-IR and HOMA-β in T2DM rats; - Improve hepatic function: ↑p-AKT (Ser473) protein levels, ↑IRS1, ↑PI3K-p85 subunit, ↑*Akt2* mRNA expression; - Mitigate hepatic lipid peroxidation: ↑ GSH and SOD activities; - Improve hIR and vascular oxidative status: ↑IRS1/PI3K/AKT2 and ↓AGE-RAGE-NOX4 pathways	Male Wistar rats	Abdelmageed et al. (2021)
Phenolic acids	PCA + GA	- PCA + GA downregulate miR-1271, improving glucose metabolism genes regulation: ↓miRNA-1271 and ↑p-IRS, p-PI3K, p-AKT, and p-FOXO1; ↑ phosphorylation of PI3K, AKT, and FOXO1 worsening by FFA; ↑*Glut2* mRNA levels	HepG2 cells (*in vitro*)	Lee and Lee (2021)
	MFA	- Attenuates the inhibitory action of miR-378b on IR/p110α, improving insulin signaling (*in vivo* and *in vitro*); - - Improve insulin sensitivity in alcoholic liver disease by modulating positively the miR-378b/PI3K-AKT pathway	Human hepatocyte L-02 cells (*in vitro*); Male C57BL/6J mice	Zhang et al*.* (2022)
Terpenes	OA	- Improves hyperglycemia and hIR: ↑p-AKT, ↓ G6P and PEPCK expression, ↓hepatic gluconeogenesis, and ↑glucose tolerance and insulin sensitivity; Improves dyslipidemia and inflammation: ↓proinflammatory interleukin levels (IL-1β, IL-6, and TNF)	Male C57BLKS/J lar-Lep^db/db^ mice	Wang et al*.* (2013)
	Celastrol	- Attenuates the deleterious effects induced by palmitate: improves glucose uptake and mitochondrial function; Cytoprotective potential via insulin signaling pathways: ↑ p-Tyr, preventing palmitate-induced IR in hepatocytes; - Potential effect on mitochondrial dysfunction and inflammation (↓NF-kB; ↓IL-8, IL-6, TNF, and CRP) against the development of hIR	C3A human liver cells (*in vitro*)	Abu Bakar et al. (2017)
	Catalpol	- Improves hIR in T2DM ameliorating AMPK/NOX4/PI3K/AKT pathway: ↓ROS mediated by NADPH oxidase type 4 (NOX4); ↑ AMPK and PI3K/AKT pathways, both *in vivo* and *in vitro*	HepG2 cells (*in vitro*); Male C57BL/6J mice	Yan et al. (2018)
Flavonoids	PSPC	- Protected against HFD-induced hIR decreasing ROS production blocking ROS-mediated ER stress: Improves glucose tolerance and insulin sensitivity: ↑GSH content and antioxidant enzymes activities; - Improves inflammations and IRS-1/PI3K/AKT signaling pathway: ↓JNK1 and IKKb/NF-kB activation; ↓GSK3β	Male ICR mice	Zhang et al. (2013)
	Morin	- Positive effect on lipogenesis: ↓serum lipid, ↓liver triglyceride levels, ↓SREBP1c, FAS, ACC, and CPT1a; - Modulates gluconeogenesis: ↑glycogen storage, ↓PEPCK and G6P protein expression; - Improves oxidative stress: ↑hepatic SOD and CAT expression; - Improves overall metabolism: ↓glycemia, insulinemia, leptinemia, MDA, IL-6 and MCP-1, and ↑serum adiponectin	HFD-induced obese mice	Naowaboot et al. (2016)
	AEd	- Positive effect on glucose metabolism: ↓serum glucose and insulin levels, ↓GRP78, PEPCK, G6P, p-PERK, p-IRE1, p-JNK, ATF4, CHOP and ↑ hepatic p-AKT; Modulates apoptosis in livers of Pb-exposed rats: ↓Bax, cytosolic cytochrome c and cleaved caspase-3, and ↑Bcl-2	Male Wistar rats	Liu et al. (2017a)
	Qu	- Attenuates serum insulin and glucose levels; - Hepatoprotective effect: ↓hIR and fibrosis, ↓ROS-associated inflammation and beneficial STAT3/SOCS3/IRS1signaling pathway regulation	Adult male Wistar rats/BDL rats	Khodarahmi et al. (2019)
Flavonoids	PG-HM	-Suppress hepatic metabolic changes through hepatic signaling pathways: leptin-insulin (AKT/FOXO1/SREBP-1c); hypoxia- inflammation (HIF-1ɑ/VEGF, TNF); mitochondrial function (complexes I–V); oxidative stress (MDA, GSH, SOD) and glycolysis/gluconeogenesis/*de novo* lipogenesis (hexokinase, phosphofructokinase, ketohexokinase, aldehyde dehydrogenase)	Male albino Wistar rats	Sharma et al. (2022)
Sapogenin and Alkaloid	FSE + trigonelline + diosgenin	- Insulin-sensitizing protective effects individually through regulation of ER stress/UPR signaling, ↓oxidative stress markers in the liver	Sprague-Dawley T2MD rats	Mayakrishnan et al. (2015)
Other bioactive compounds	LA + CLA + B9	- Improves lipid metabolism, inflammation, and oxidative stress; Alters gut microbiota composition; Anti-inflammatory effects: ↓TNF secretion	Clinical trial (obese Chinese women aged 36–66 years)	Chen et al. (2019)
	GFNP	-Improves hIR through IRS2/PI3K/AKT signal pathway: ↑*Akt2, Irs2*, and *Pi3k* mRNA expressions; ↓gluconeogenesis (AKT/FOXO1/PGC-1α pathway) and ↑glycogenesis (AKT/GSK3β pathway) in the liver	db/db Diabetic mice	Li et al. (2019)
	S1P	- Improves hIR by reducing mitochondrial ROS generation: ↓ROS production, ↑glucose uptake and insulin signaling	Normal human hepatocyte cell line LO2 (*in vitro*)	Fang et al. (2020)
	Lf	-Improves hIR and pancreatic dysfunction; Decreases serum concentrations of glycated protein, insulin, cholesterol, and triacylglycerol; Increases liver insulin sensitivity: ↑InsR, IRS-1, PI3K, and AKT proteins expressions	T2DM mice	Du et al. (2022)

SOL, *sonchus oleraceus linn*; AMBE, *aronia melanocarpa* (Michx.) *elliott* berry extract; RSV, resveratrol; FA, ferulic acid; EGCG, epigallocatechin-3-O-gallate; PCA, protocatechuic acid; GA, gallic acid; MFA, methyl ferulic acid; OA, oleanolic acid; PSPC, purple sweet potato color; AEd- Epigallocatechin-3-gallate dimer; Qu, quercetin; BDL, bile duct ligation; PG-HM, psidium guajava L. (Myrtaceae); FSE, fenugreek (*Trigonella foenum graecum*) seed; LA, lactic acid; CLA, conjugated linoleic acid; B9, folate; GFNP, amino-acid-funcitionalized gadofullerene nanoparticles; S1P, shingosine-1-phosphate; Lf, lactoferrin.

### Phytochemicals and hepatic insulin resistance: potential mechanisms involved in insulin signaling pathway

**FIGURE 3:**
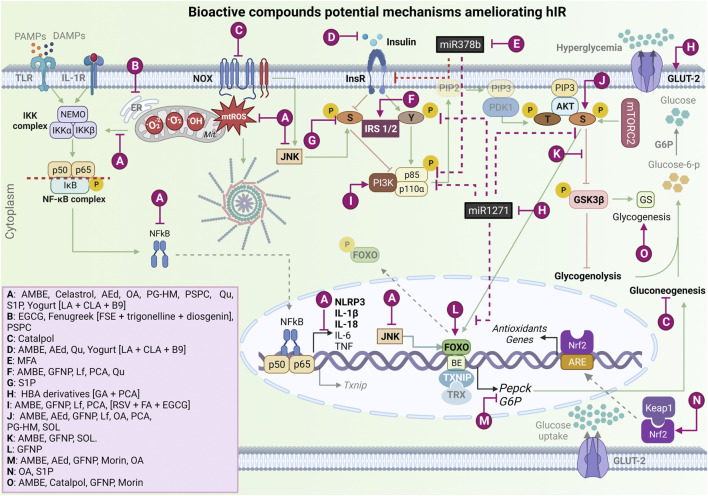
Bioactive compound potential mechanisms improve hepatic insulin resistance and ameliorate oxidative stress and NLRP3 inflammasome activation. Ⓐ Bioactive compounds that can decrease mtROS production and mtROS-associated inflammation, downregulating NF-kB complex and JNK activation, *in vitro* and T2DM/hIR *in vivo* models [AMBE (Mu et al., 2020); Celastrol (Abu Bakar et al., 2017); AEd (Liu C.-M. et al., 2017); OA (Wang et al., 2013); PG-HM (Sharma et al., 2022); PSPC (Zhang et al., 2013); Qu (Khodarahmi et al., 2019); S1P (Fang et al., 2020); and Yogurt metabolites [LA + CLA + B9] (Chen et al., 2019)]. Ⓑ Phytochemicals that act on ER stress *in vitro* and T2DM/hIR *in vivo* models [AEd; Fenugreek extract [FSE + trigonelline + diosgenin] (Mayakrishnan et al., 2015), PSPC]. Ⓒ Phytochemical that has a suppressive effect on NOX4 overexpression in *in vitro* and T2DM/hIR *in vivo* models [Catalpol (Yan et al., 2018)]. Ⓓ Phytochemicals that decrease hyperinsulinemia *in vitro* and *in vivo* T2DM model [AMBE, AEd, Qu, Yogurt metabolites [LA + CLA + B9]. Ⓔ Phytochemical that downregulates miR-378b and increases InsR, *in vitro* and *in vivo* hIR alcohol-induced model. [MFA (Zhang et al., 2022)]. Ⓕ Bioactive compounds that upregulate the InsR proteins expressions in T2DM/hIR *in vivo* model [AMBE, GFNP (Li et al., 2019), Lf (Du et al., 2022), PCA (Abdelmageed et al., 2021), Qu]. Ⓖ Bioactive compound that downregulates p-IRS-1/2 in serine in hIR *in vitro* model [S1P (Fang et al., 2020)]. Ⓗ Phytochemical that downregulates miRNA-1271, increases InsR, and stimulates GLUT-2 expression in T2DM *in vitro* model [HBA derivatives [GA + PCA] (Lee and Lee, 2021)]. Ⓘ Phytochemicals that activate PI3K *in vitro* and in T2DM/hIR *in vivo* models [AMBE, GFNP, PCA, (RSV + FA + EGCG) (Kang and Chiang, 2022)]. Ⓙ Bioactive compounds that activate AKT *in vitro* and in T2DM/hIR *in vivo* models [AMBE, AEd, GFNP, Lf, OA, PCA, PG-HM, SOL (Chen et al., 2020)]. Ⓚ Bioactive compounds that inhibit GSK3β activation *in vitro* and in T2DM/hIR *in vivo* [AMBE, GFNP, SOL]. Ⓛ Bioactive compound that increases FOXO phosphorylation in T2DM/hIR *in vivo* model [GFNP]. Ⓜ Bioactive compounds that decrease G6P and PEPCK expression *in vitro* and *in vivo* models [AMBE, AEd, GFNP, Morin (Naowaboot et al., 2016), OA)]. Ⓝ Bioactive compounds that upregulate Nrf2 expression and its nuclear translocation *in vitro* and in T2DM/hIR *in vivo* models [OA, S1P]. Ⓞ Bioactive compounds that hepatic glycogenesis *in vitro* and *in vivo* models [AMBE, Catalpol, GFNP, Morin]. mtROS, mitochondrial reactive oxygen species; NF-κB, nuclear factor kappa beta; JNK, Jun N-Terminal Kinase; T2DM, type 2 mellitus diabetes; hIR, hepatic insulin resistance; AMBE, *Aronia melanocarpa* (Michx.) *Elliott* berry extract; AEd, A-type dimeric epigallocatechin-3-gallate (A-type-EGCG-dimer); OA, Oleanoic Acid; PG-HM, *Psidium guajava L.* (*Myrtaceae*); PSPC, Purple Sweet Potato Color; Qu, Quercetin; S1P, Sphingosine-1-phosphate; LA, lactic acid; CLA, Conjugated Linoleic Acid; B9, folate; ER, endoplasmic reticulum; FSE, fenugreek seed extract; NOX, nicotinamide adenine dinucleotide phosphate oxidase; miR, microRNA; InsR, insulin receptor; MFA, Methyl Ferulic Acid; GFNP, Amino-Acid-Functionalized Gadofullerene Nanoparticles; Lf, Lactoferrin; PCA, Protocatechuic acid; IRS1/2, Insulin receptor substrate; GLUT-2, glucose transporter 2; HBA, Hydroxybenzoic Acid Derivatives; GA, Gallic Acid; PI3K, phosphoinositide-3-kinase enzyme; RSV, Resveratrol; FA, Ferulic Acid; EGCG, epigallocatechin-3-gallate; SOL *Sonchus oleraceus* Linn; GSK3β, glycogen synthase kinase-3β; AKT, protein kinase B (PKB); FOXO, forkhead box; G6P, glucose 6-phosphatase; PEPCK, phosphoenolpyruvate carboxykinase; Nrf2, nuclear factor erythroid 2-related factor 2; IL-1β, interleukin 1β; Y, tyrosine; GS, glycogen synthase; IKKβ, IkappaB kinase β. → Green arrow; upregulation/stimulation. ⊣ Red blunted arrow; downregulation/inhibition. ⇢ Black dotted arrow; translocation to/from nucleus. ↳ gene expression. → Purple arrow; upregulation/stimulation. ⊣ Purple blunted arrow; downregulation/inhibition. Created with BioRender.com.

Regarding the insulin signaling pathway, some works have involved a phenolic extract or combined with phytochemicals to ameliorate hIR. Chen et al. (2020) was the first to evaluate the effects of phenolic compounds in *Sonchus oleraceus* Linn (SOL) extract on hIR in diabetic rats and HepG2 cells. The authors orally administered three concentrations of SOL (100, 200, or 400 mg/kg/day) in the male Sprague-Dawley rats and treated HepG2 cells with 200 μg/mL SOL extract. They found that in the diabetic rats treated with SOL extract, there was a positive effect on glucose homeostasis due to the downregulation of AMPK/AKT/GSK3β pathways, suggesting that the phenolic compounds present in the extract may have regulated AMPK and AKT phosphorylation and inhibited the activation of GSK3β, improving hIR (Figure 3). Regarding the HepG2 cells, AMPK, AKT, and GSK3β displayed consistent transcript regulation. Thus, they suggested that SOL extract at 400 mg/kg/day for 6 weeks had a metformin-like effect in diabetic rats, owing to the positive regulation of the insulin signaling pathway (Chen et al., 2020).

As aforementioned, a combination of phytochemicals has also been proposed to ameliorate muscle and hIR because these compounds may have complementary and synergistic properties that might be more effective than antidiabetic drugs, such as metformin and 5-aminoimidazole-4-carboxamide ribonucleotide (AICAR), in improving IR. Kang and Chiang (2022) tested *in vitro* (L6 cell line, HepG2 cells and male C57BL/6 muscle primary culture) the efficiency of a formula consisting of resveratrol (10 mM), ferulic acid (25 mM), and epigallocatechin-3-O-gallate (5 mM) and demonstrated that the phenolic combination systemically regulated glucose metabolism, enhancing GLUT4 translocation to the plasma membrane in insulin-resistant muscle cells, regulating insulin-independent (calcium and AMPK) and insulin-dependent (PI3K) signaling molecules. Moreover, the tested formula increased glycogen synthesis and decreased glucose production by insulin-resistant liver cells, with antidiabetic potential in the myotubes, hepatocytes, and adipocytes due to their lipid content reduction. Although these three phenolic compounds might act in distinct pathways, they are able to enhance insulin-dependent and independent vesicle trafficking and glucose transport mechanisms, improving carbohydrate and lipid metabolism and thus ameliorating the muscle and hIR (Figure 3) (Kang and Chiang, 2022).

### Phytochemicals and insulin signaling pathway: potential mechanisms involved in ameliorating oxidative stress and inflammation

Most studies have considered the antioxidant and anti-inflammatory role of bioactive compounds in exploring alterations in the PI3K/AKT signaling pathway. Terpenoids and phenolic compounds, such as flavonoids, anthocyanins, catechins, phenolic acids, and alkaloids are examples of phytochemicals involved in ameliorating hIR, acting mainly in mechanisms underlying ROS generation and inflammatory pathways.

Regarding terpenoids, our search retrieved four articles on the effects of oleanolic acid, celastrol, catalpol, and saponins on oxidative stress and inflammation, which are discussed in the following paragraphs. Oleanolic acid (OA) is a natural triterpenoid with antioxidant activity and a protector effect against oxidative stress in HepG2 cells (Wang et al., 2013). Wang et al. (2013) aimed to determine the OA effects (20 mg/kg/day, i.p., for 2 weeks) on hIR in Lep^db/db^ obese diabetic mice. They demonstrated that OA improves hyperglycemia and hIR in mice, based on the treatment being able to increase AKT phosphorylation, decreasing G6P and phosphoenolpyruvate carboxykinase (PEPCK) expression, inhibiting hepatic gluconeogenesis, and improving glucose tolerance and insulin sensitivity. Moreover, they suggested that this improvement is a result of mitochondrial ROS inhibition owing to the OA treatment, which resulted in GSH increases and GSSG, as well as stabilized the Nrf2 and glutathione cysteine ligase catalytic subunit protein expression, protecting Lep^db/db^ obese diabetic mice against severe mitochondrial dysfunction. Finally, OA treatment improved dyslipidemia and inflammation, reducing proinflammatory interleukin levels, such as those of IL-1β, IL-6, and TNF (Figure 3) (Wang et al., 2013).

Celastrol, a pentacyclic-triterpene derived from the root of *Tripterygium wilfordii Hook F*., was reported to mitigate IR and inflammation in experimental disease models (Abu Bakar et al., 2017). However, its mechanistic actions were unclear until Abu Bakar et al. (2017) conducted an *in vitro* study to understand celastrol’s role in palmitate-induced IR in C3A human hepatocytes. The hepatocytes that received palmitate (0.75 mM for 48 h) had mitochondrial dysfunction and IR, as well as upregulation in NF-κB and JNK signaling pathways with an increase in proinflammatory cytokine release. Moreover, palmitate altered the phosphorylated Tyr-612 and Ser-307 of IRS-1 proteins in hepatocytes, resulting in IR. By contrast, celastrol (30 nM) attenuated the deleterious effects induced by palmitate, leading to an improvement in glucose uptake and mitochondrial function, attenuating the sustained activation of NF-kB and downregulating the release of proinflammatory cytokines (IL-8, IL-6, TNF, and CRP). Likewise, celastrol recovered the phosphorylation in the Tyr-602 residue, preventing palmitate-induced IR in hepatocytes (Figure 3). Thus, the authors demonstrated that celastrol has cytoprotective potential via insulin signaling pathways, suggesting the potential effect on mitochondrial dysfunction and inflammation against the development of hIR (Abu Bakar et al., 2017).

Another study evaluated the effect of the iridoid glucoside catalpol on hIR in T2DM. Yan et al. (2018) demonstrated that catalpol treatments improved hepatic oxidative stress mediated by NADPH oxidase type 4 (NOX4) and activated hepatic AMPK and PI3K/AKT pathways, *in vivo* (male C57BL/6J mice treated with 100 or 200 mg/kg/d) and *in vitro* (*HepG2 cells incubated with 20, 40 or 80 μM catalpol*). *In vitro*, the catalpol treatment prevented gluconeogenesis and increased glycogen synthesis in HepG2 cells. Moreover, the suppressive effect of catalpol on glucosamine-induced NOX4 overexpression was reduced (Figure 3) by AMPK knockdown with short interfering RNA (siRNA). They suggested that catalpol ameliorates hIR in T2DM by acting on AMPK/NOX4/PI3K/AKT pathway (Yan et al., 2018).

Regarding phenolic compounds, our search retrieved eight articles reporting the effects of flavonoids, anthocyanins, phenolic acids, alkaloids, and proanthocyanidins dimer on oxidative stress and inflammation, discussed in the following paragraphs.

Quercetin (Qu) is one of the most studied flavonoids; this well-known antioxidant has anti-inflammatory, antifibrotic, and antidiabetic properties. Thus, the use of Qu as an adjunctive treatment was shown to prevent liver damage. Notably, diabetes mellitus and cirrhosis are interconnected in a condition called hepatogenous diabetes, a prevalent hepatic fibrosis complication. In this manner, IR and inflammatory mediators are correlated with hepatic fibrosis, and the gold-standard model used to study this condition is bile duct ligation (BDL) (Khodarahmi et al., 2019). Thus, the study conducted by Khodarahmi et al. (2019) aimed to determine whether Qu (30 mg/kg/day) improves hIR and hepatic fibrosis in BDL in adult male Wistar rats, an increased oxidative stress was observed. The Qu treatment attenuated the increase in serum insulin and glucose levels and had a hepatoprotective effect. The antidiabetic effect of Qu was associated with a downregulation in the mRNA and protein levels of STAT3 and SOCS3 and jointly occurred with an IRS1 upregulation. Thus, the authors suggested that Qu ameliorates hIR and fibrosis by inhibiting ROS-associated inflammation and promoting STAT3/SOCS3/IRS1signaling pathway regulation (Figure 3) (Khodarahmi et al., 2019).

Additionaly, Sharma et al. (2022) have evaluated the potential of the hydroethanolic extract of leaves of *Psidium guajava L. (Myrtaceae)* (PG-HM), commonly known as guava, to suppress the alterations in the hepatic molecular signals due to unrestricted fructose (15%) drinking by growing rats. Notably, guava leaves have medicinal effects attributed to Qu, their major phytoconstituent (Sharma et al., 2022). In this context, the authors used male Wistar rats (4 weeks old) in control groups with access to fructose drinking solution (15%) for 4 or 8 weeks (i.e., until puberty or early adulthood), respectively, and the treated groups additionally received PG-HM (500 mg/kg, p.o.). They reported that the PG-HM suppressed hepatic metabolic changes through the hepatic signaling pathways of 1) leptin-insulin (AKT/FOXO1/SREBP-1c); 2) hypoxia-inflammation (HIF-1ɑ/VEGF, TNF); 3) mitochondrial function (complexes I–V); 4) oxidative stress (MDA, GSH, superoxide dismutase (SOD) and 5) glycolysis/gluconeogenesis/*de novo* lipogenesis (hexokinase, phosphofructokinase, ketohexokinase, aldehyde dehydrogenase) (Figure 3) (Sharma et al., 2022). Similarly, the insulin-sensitizing effect of PG-HM and its ethyl acetate fraction was elucidated using HepG2 cells grown in media enhanced with fructose. Moreover, in murine hepatocytes cultured in fructose-rich media, PG-HM (35 µg mL^−1^) outperformed pioglitazone (15 µM) and metformin (5 mM) to suppress hIR. Bioactive polyphenols in guava leaves were demonstrated to play an important role in the prevention and treatment of high carbohydrate and HFD-induced hepatic steatosis and dyslipidemia. In that study, the ingestion of fructose (15%) from the weanling stage led to hepatic injury, but PG-HM successfully suppressed all the hepatic steatosis and dyslipidemia. Thus, this study established that PG-HM has the potential to suppress hepatic metabolic alteration for the management of energy homeostasis in juveniles and young adults (Sharma et al., 2022).

Another flavonoid compound, morin, found in the *Moraceae* family’s members, has antioxidant and anti-inflammatory properties in hIR. Thus, Naowaboot et al. (2016) investigated the effect of morin (50 or 100 mg/kg/day once daily for 6 weeks) on hIR, oxidative stress, and inflammation in HFD-induced obese mice (60% kcal from fat) (Naowaboot et al., 2016). They showed morin administration had a positive effect on lipogenesis, decreasing serum lipid and liver triglyceride levels; downregulating SREBP1c, FAS, and ACC; and upregulating carnitine palmitoyltransferase 1a (CPT1a). Morin also interfered with gluconeogenesis, stimulating glycogen storage and repressing PEPCK and G6P protein expression (Figure 3), and affected oxidative stress activities, increasing hepatic SOD and catalase (CAT) expression after treatment. Moreover, they found a decrease in glycemia, insulinemia, leptinemia, MDA, IL-6, and monocyte chemoattractant protein-1 (MCP-1) and an increase in serum adiponectin. These findings suggest the positive effect of morin in HFD-induced obesity by lipogenesis, gluconeogenesis, inflammation, and the suppression of oxidative stress activities (Naowaboot et al., 2016).

*Aronia melanocarpa* (Michx.) Elliott berry extract (AMBE) is rich in polyphenolic compounds, such as anthocyanins, flavonoids, and phenolic acids, and its antioxidant and hypoglycemic effects have been described in animal models. Mu et al. (2020) examined the effects of AMBE supplementation (100 and 400 mg/kg body weight daily for 8 weeks) on hIR HFD and STZ-induced T2DM rats (Mu et al., 2020). The AMBE improved glucose tolerance, decreasing glycemia and insulinemia, and had a positive impact on the HOMA-IR (homeostatic model assessment for insulin resistance) index. Moreover, in a dose-dependent manner, AMBE increased the activities of glucokinase and pyruvate kinase enzymes, raising the level of hepatic glycogen in animals with T2DM and significantly reducing the activities of the enzymes responsible for hepatic gluconeogenesis, such as PEPCK and G6P (Figure 3). Furthermore, AMBE improved hepatic function, decreasing lipid accumulation, oxidative stress, and inflammation in the T2DM liver. Thus, AMBE reduced ROS production and MDA levels and increased GSH activity. They also observed a downregulation in hepatic mRNA of *Il-1b, Il-6*, and *Tnf* expression. The authors described that AMBE attenuated hIR through the regulation of the PI3K/AKT signaling pathway, with an upregulation of p-IRS-2, p-PI3K, p-AKT, p-GSK3β, and GLUT2 (Figure 3), supporting the use of AMBE as a functional food additive because the berry extract may promote glycogen synthesis and improve hIR in T2DM (Mu et al., 2020).

Regarding anthocyanins, purple sweet potato color (PSPC) has strong antioxidant, anti-inflammatory, neuro, and hepatoprotective effects. In this context, Zhang et al. (2013) showed that male mice treated with 700 mg/kg/day of PSPC protected against HFD-induced hIR, decreasing ROS production, and blocking ROS-mediated ER stress (Figure 3) (Zhang et al., 2013). They demonstrated that PSPC restored glucose tolerance and insulin sensitivity by suppressing ROS generation and because of an improvement in GSH content and antioxidant enzyme activities. Moreover, the compound inhibited JNK1 and IKKb/NF-kB activation (Figure 3). Regarding the insulin signaling pathway, PSPC acted in the inactivation of GSK3β, improving the IRS-1/PI3K/AKT signaling pathway (Figure 3). Their study reinforced that PSPC may suppress hyperglycemia by inhibiting alpha-glucosidases and attenuate oxidative stress, inflammation, and apoptosis in mouse hepatocytes, contributing to the improvement in hIR (Zhang et al., 2013).

Protocatechuic acid (PCA) is a phenolic acid found in many plants. Researchers have demonstrated its hypoglycemic, anti-inflammatory, and antioxidant effects, improving cardiac dysfunction and redox status, as well as stimulating glucose metabolism in skeletal muscles, in T2DM rats (Abdelmageed et al., 2021). However, PCA effects on hIR have not been fully investigated. Abdelmageed et al. (2021) postulated that PCA would modulate the IRS1/PI3K/AKT2 pathway, improving hIR, and AGE-RAGE-NOX4 signaling in HFD, high fructose (25% w/v), and STZ (35 mg/kg, i.p)-induced T2DM rats (treated with PCA 100 mg/kg/day, orally) (Abdelmageed et al., 2021). The authors demonstrated that PCA treatment was able to reduce fasting glycemia and insulinemia, improving HOMA-IR, HOMA-β, and insulinogenic indexes in T2DM rats. In addition, PCA improved hepatic function, increasing p-AKT (Ser473) protein levels, IRS1, PI3K-p85 subunit (Figure 3), and *Akt2* mRNA expression in the liver and mitigated hepatic lipid peroxidation, restoring GSH and SOD activities to near normal concentrations. Moreover, PCA has ameliorated aortic oxidative stress owing to the reduction in advanced glycation end products’ (AGEs’) levels and downregulating the vascular expression of RAGE (AGE receptors) and *Nox4* mRNA. Their results show that PCA improved hIR and vascular oxidative status by modulating the IRS1/PI3K/AKT2 and AGE-RAGE-NOX4 pathways, respectively, proving their hypothesis (Abdelmageed et al., 2021).

Fenugreek (*Trigonella foenum graecum*) seed, a traditional medicinal herb, has some phytoconstituents with known hypoglycemic and hypolipidemic properties (Mayakrishnan et al., 2015); however, the mechanism involved in the insulin signaling pathway requires further research. In this regard, Mayakrishnan et al. (2015) assessed the protective effects of fenugreek seed extract (FSE, 300 mg/kg) and its alkaloids trigonelline (40 mg/kg) and diosgenin (60 mg/kg) on hIR. They hypothesized that FSE and its phytoconstituents act through ER stress/UPR signaling and the related oxidative stress in the liver of Sprague-Dawley T2DM rats [HFD-induced (58% fat) with low-dose STZ (35 mg/kg)]. They observed that T2DM rats had an increase in ER chaperones Bip, protein disulfide isomerase, and ER stress-associated proapoptotic markers [C/EBP homologous protein (CHOP), Caspase12, and Caspase3] in the liver, an increase in lipid peroxidation and a decrease in antioxidant levels. In conclusion, they demonstrated that FSE, trigonelline, and diosgenin had insulin-sensitizing protective effects individually through the regulation of ER stress/UPR signaling because, in addition to biochemical markers, FSE and its phytoconstituents improved the expression levels of ER stress/UPR pathway proteins and the levels of the oxidative stress markers in the liver (Figure 3) (Mayakrishnan et al., 2015).

The high bioactive molecule content in persimmon (*Diospyros kaki L.*) fruit has been shown to have health benefits, especially for metabolic diseases such as hyperlipidemia, hyperglycemia, and oxidative stress. Proanthocyanidin, carotenoids, tannins, flavonoids, and catechin are found in persimmon fruits, and a new proanthocyanidins dimer was discovered in persimmon fruits: the A-type EGCG (epigallocatechin-3-gallate) dimer (AEd). This AEd was shown to have potent anti-amyloidogenic substance and inhibit adipogenesis. Notably, lead (Pb) exposure may alter glucose metabolism and result in hyperlipidemia, hyperglycemia, and IR in rats (Liu C.-M. et al., 2017). Thus, Liu C.-M. et al. (2017) conducted a study to investigate whether AEd (200 mg/kg body weight/day, intragastrically for 3 months) inhibited Pb-induced IR and apoptosis in the liver by inhibiting hepatic ER stress in male Wistar rats (0.05% w/v Pb acetate in their drinking water). For comparison purposes, the authors used one drug with hypoglycemic action (pioglitazone). They found that AEd supplementation similarly decreased serum glucose and insulin levels, downregulating the expression levels of glucose-regulated protein 78 (GRP78), PEPCK, G6P, phosphorylated protein kinase RNA (PKR)-like ER kinase (p-PERK), phosphorylated inositol-requiring enzyme (p-IRE1), p-JNK, activating transcriptional factor 4 (ATF4), and CHOP, and upregulating hepatic p-AKT in the Pb group (Figure 3). Moreover, AEd prevented Pb-induced ER stress and apoptosis in livers, reducing ROS production and restoring the SOD and GPx activities. Regarding apoptosis, AEd downregulated Bax (Bcl-2 associated X-protein), cytosolic cytochrome c, and cleaved caspase-3 and upregulated Bcl-2 in livers of Pb-exposed rats. Their results suggested that AEd may be a natural candidate for hIR prevention in rats exposed to Pb, and its protective effects might be, at least in part, associated with the inhibition of ER stress and apoptosis in the liver (Liu C.-M. et al., 2017).

#### Phenolic compounds and miRNA: potential mechanisms involved in improving hepatic insulin resistance

MiRNAs are small non-coding RNAs involved in several biological processes because they bind to the 3′ region of their target mRNA; some of them modulate protein expressions involved in glucose metabolism and homeostasis. MiR-378b, miR-26a, miR-125a, miR206, miR-223, miR1271, miR-29a, and miR-451 are related to the hepatic insulin signaling pathway, and the modulation of the IRS/PI3K/AKT/FOXO1 pathway by miRNAs has been investigated as a mechanism through which phytochemicals improve insulin signaling (Lee and Lee, 2021; Zhang et al., 2022).

Lee and Lee (2021) investigated the antidiabetic activity of hydroxybenzoic acid derivatives [PCA and gallic acid (GA)] through the downregulation of miRNA-1271 and upregulation of its targets, such as p-IRS, p-PI3K, p-AKT, and p-FOXO1, improving the regulation of glucose metabolism genes (Figure 3). The HepG2 cells treated with FFA and then exposed to hydroxybenzoic acid derivatives ameliorated the phosphorylation of PI3K, AKT, and FOXO1 worsened by FFA, and GA increased *Glut2* mRNA levels (Figure 3). Moreover, miR-1271 was involved in the protective effect of GA against hIR, using miR-1271 mimic or miR-1271 inhibitor, revealing a possible mode for phytochemicals to improve hIR (Lee and Lee, 2021).

Another microRNA described as an important regulator of hepatic insulin signaling is miR-378b. It has universal expression in some tissues, and its overexpression is related to impaired glucose metabolism because miR-378b targets InsR and p110α (Zhang et al., 2022). Zhang et al. (2022) evaluated the influence of methyl ferulic acid (MFA) on insulin sensitivity in ethanol-induced *in vitro* (human hepatocyte L-02 cells, cultured with MFA 100, 50, and 25 µM) and in alcohol-fed male C57BL/6J mice [high-dose of MFA (20 mg/kg), medium-dose of MFA (10 mg/kg) and low-dose of MFA (5 mg/kg)] and provided insights into the miR-378b-mediated PI3K/AKT pathway in hIR. They found that MFA downregulated miR-378b and increased InsR and p110α expressions, corroborating this effect by the overexpression and inhibition of miR-378b (Figure 3). Thus, MFA probably positively modulates the miR-378b/PI3K-AKT pathway, improving insulin sensitivity in alcoholic liver disease by attenuating the inhibitory action of miR-378b on IR/p110α, activating insulin signaling *in vivo* and *in vitro*, alleviating alcohol-induced hIR (Zhang et al., 2022).

#### Bioactive compounds and insulin signaling pathway: potential mechanisms involved in improving hepatic insulin resistance

Although the scope of this review was the ameliorative role of phytochemicals in NLRP3 inflammasome activation and oxidative stress improving hIR, our searches retrieved three articles related to bioactive compounds worth reviewing. They are described in this section.

Sphingosine-1-phosphate (S1P) is a bioactive lipid that plays a role in many biological processes. It is metabolized from membrane sphingolipids, specifically sphingomyelin, by the enzyme sphingosine kinase 1 (SphK1). An increase in serum concentrations of S1P levels was related to being positively correlated with insulin-stimulated glucose uptake, suggesting that extracellular S1P may affect the insulin signaling pathway. Moreover, S1P levels affected ROS production in many cell types. Despite the promising results, the role of S1P in insulin signaling has been controversial because the literature has shown that palmitate-stimulated hepatocytes had an increase in S1P and, consequently, an insulin signaling impairment (Fang et al., 2020). In this regard, Fang et al. (2020) investigated the S1P mechanism on ROS generation and hIR. To achieve that objective, they treated normal human hepatocyte cell line LO2 with 1,000 nM insulin (48 h) and observed a decrease in glucose uptake (Figure 3), an increase in serine phosphorylation of IRS, ROS accumulation in the cytosol and mitochondria, and upregulation of Nrf2 expression and nuclear translocation. However, S1P treatment (0.1–5.0 μM) reversed the effects on ROS production, glucose uptake, and insulin signaling (Figure 3). Moreover, they demonstrated that H_2_O_2_ had the most significant inhibitory role, reversing the beneficial effects of S1P in ameliorating hIR (Fang et al., 2020). Thus, they concluded that S1P can improve hIR by reducing mitochondrial ROS generation, and the possible mechanism may be involved in H_2_O_2_ signaling (Gurzov et al., 2014; Fang et al., 2020; Joseph et al., 2020; Zhang et al., 2022).

Regarding bioactive compounds in milk and its derivatives, we found two original works suggesting their potential benefits of ameliorating hIR (Chen et al., 2020; Du et al., 2022). The first paper was that of Du et al. (2022). They demonstrated the lactoferrin (Lf) supplementation effect on T2DM mice. Lf is an iron-binding glycoprotein, and T2DM mice supplemented with 0.5% or 2% Lf solution for 12 weeks had improvements in hIR and pancreatic dysfunction. Moreover, they observed changes in serum concentrations of glycated protein, insulin, cholesterol, and triacylglycerol, and an increase in liver insulin sensitivity, suggesting that 0.5% or 2% Lf solution for 12 weeks improved pancreatic dysfunction by reducing oxidative stress and inflammation responses. Moreover, Lf supplementation upregulated the InsR, IRS-1, PI3K, and AKT protein expressions in the liver (Figure 3) (Du et al., 2022).

The second study demonstrated that the daily intake of 220 g of conventional yogurt, fermented by *Lactobacillus delbrueckii* ssp. *bulgaricus* and *Streptococcus thermophiles*, improved IR and liver fat (Chen et al., 2019). Chen et al. (2019) conducted a randomized controlled trial with women aged from 36 to 66 years who were obese and lived in China. They suggested that lactic acid, conjugated linoleic acid (CLA), and folate in yogurt improved lipid metabolism, reducing inflammation and oxidative stress and altering the gut microbiota composition (Chen et al., 2019). Notably, lactic acid and CLA have anti-inflammatory effects due to TNF secretion suppression (Figure 3), and CLA increased GSH synthesis without lipoperoxidation. Moreover, *S. thermophiles* may synthesize folate during fermentation, and this B vitamin has an anti-inflammatory effect, decreasing fasting insulin and ameliorating hIR. Thus, yogurt might ameliorate IR through anti-inflammatory and antioxidative stress effects on the insulin signaling pathway and islet cells (Chen et al., 2019).

Notably, numerous bioactive compounds are proteinaceous, such as peptides, peptide derivatives, amino acid-like components, and amino acids. Thus, another type of antidiabetic strategy found in literature was the administration of amino-acid-functionalized gadofullerene nanoparticles (GFNPs) intraperitoneal. GFNPs are derived from amino acids and can eliminate ROS, protect mitochondria against damage related to oxidative stress, and reduce inflammatory reactions (Li et al., 2019). Li et al. (2019) improved hIR via intraperitoneal administration of GFNPs (1.5 μmol/kg/d; 3 μmol/kg/d; 6 μmol/kg/d) in db/db diabetic mice, through the activation of IRS2/PI3K/AKT signal pathways, inhibiting gluconeogenesis and increasing glycogenesis in the liver. This phenomenon occurs because, notably, when mRNA expressions of genes of the main signaling molecules related to hIR (MAPK, PPARα, and PPARγ) were evaluated in diabetic mice, there was no difference between those treated with amino acid derivatives and those treated with saline solution, showing that these were not the potential targets of GFNPs. *Akt2, Irs2*, and *Pi3k* mRNA expressions were upregulated by GFNPs, indicating activation of the IRS/PI3K/AKT pathway. Furthermore, these compounds inhibit gluconeogenesis and promote hepatic glycogenesis by regulating the AKT/FOXO1/PGC-1α AND AKT/GSK3β pathways (Figure 3). Additionally, highly accumulated in the pancreas and liver, GFNPs relieved hepatic steatosis in the liver, maintaining systemic glucose and lipid metabolic homeostasis without obvious toxicity. Together, GFNPs reverse the dysfunctions of the pancreas and improve hIR, providing a promising approach for T2DM treatment (Li et al., 2019).

## Conclusion

As we have presented in this review, there is evidence linking the activation of the NLRP3 inflammasome and its correlated pathways, involving mtROS and other activating signals, to hIR progression. In this line of thinking, various phytochemicals and bioactive compounds have been proposed as anti-inflammatory agents as complementary or alternative anti-inflammatory therapeutics in treating chronic inflammatory disorders, such as hIR. Moreover, there is a consensus in literature consensus regarding the *in vivo* and *in vitro* effects of bioactive compounds on hIR: they demand further investigation and clinical studies are required to confirm the findings and provide insights into the underlying mechanisms of action. Thus, these compounds are suitable for use as an additional therapeutic strategy to improve hIR and correlated conditions. Furthermore, our narrative review also reveals topics for further research to search for the synergistic actions of these bioactive compounds as adjunctive therapy to improve the treatment of several diseases involving oxidative stress and inflammation, such as hIR.

## Abbreviations

AKT, protein kinase B; AMPK, AMP-activated protein kinase; ASC, apoptosis-associated speck-like protein; DAMPs, damage-associated molecular patterns; FA, free fatty acids; FOXO, forkhead box protein O; G6P, glucose 6-phosphatase; GSK3, glycogen synthase kinase 3; GSDMD, gasdermin D; hIR, hepatic insulin resistance; IL-1R, interleukins receptors; InsR, insulin receptor; IRS, insulin receptor substrates; JAK/STAT, janus kinase/signal transducers and activators of transcription; JNK, c-Jun N-terminal protein kinase; MAPK, mitogen-activated protein kinase; miRNAs, microRNAs; mtROS, mitochondrial reactive oxygen species; NF-κB, nuclear factor-κB; NLRP3, NLR family pyrin domain containing 3; NOX, NADPH Oxidase; Nrf2, nuclear factor erythroid 2-related factor 2; ox-mtDNA, oxidized mitochondrial DNA; PAMPs, pathogen-associated molecular patterns; PEPCK, phosphoenolpyruvate carboxykinase; PI3K, phosphatidylinositol 3-kinase; PIP3, phosphatidylinositol (3,4,5)-trisphosphate; ROS, reactive oxygen species; TLRs, Toll-Like receptor family; TXNIP, thioredoxin interacting protein.
